# Surgical Strategies and Outcomes for Intracranial Chondromas: A Retrospective Study of 17 Cases and Systematic Review

**DOI:** 10.3389/fonc.2022.865865

**Published:** 2022-05-26

**Authors:** Hongyuan Liu, Qing Cai, Junting Li, Yafei Xue, Yunze Zhang, Zongping Li, Tianzhi Zhao, Yingxi Wu

**Affiliations:** ^1^ Department of Neurosurgery, Tangdu Hospital, Air Force Medical University, Xi’an, China; ^2^ Department of Neurosurgery, Mianyang Central Hospital, School of Medicine, University of Electronic Science and Technology of China, Mianyang, China; ^3^ Department of Pathology, Tangdu Hospital, Air Force Medical University, Xi’an, China

**Keywords:** intracranial chondromas, surgical approach, endoscopic endonasal transsphenoidal approach, imaging features, prognosis

## Abstract

**Objective:**

To improve the diagnosis and treatment of intracranial chondromas (ICDs) by discussing the clinical manifestations and imaging characteristics of ICDs, as well as surgical methods and treatment strategies.

**Methods:**

We retrospectively analyzed 17 patients diagnosed with ICDs who underwent microsurgery or endoscopic transsphenoidal surgery at the Tangdu Hospital of Air Force Military Medical University and the Mianyang Central Hospital from January 2010 to November 2021. Clinical manifestations, imaging examinations, surgical treatments, and prognosis of these patients were analyzed.

**Results:**

ICDs had often been misdiagnosed as craniopharyngioma, chordoma, schwannoma, cavernous hemangioma, pituitary adenoma, and meningioma before surgery. Of the 17 cases, gross total resection (GTR) was performed in 10 cases, subtotal resection (STR) in 5, and partial resection in 2. GTR of tumor was achieved in eight cases *via* the endoscopic endonasal transsphenoidal approach (EETA) or the extended endoscopic endonasal transsphenoidal approach (EEETA), and the remaining patients underwent craniotomies. Clinical symptoms were assessed 1 week after surgery, 10 cases were relieved at varying degrees, and four cases had no improvement. Postoperative complications included right-limb hemiparesis, diplopia, eyelid ptosis, pulmonary infection, subcutaneous hydrops, cerebrospinal-fluid leakage (CSFL), and intracranial infection (ICI). One patient received gamma knife treatment at 3 months after surgery, two patients died due to tumor progression, and the remaining patients had no tumor recurrence.

**Conclusions:**

ICDs lack typical imaging features and are often misdiagnosed. The EETA or EEETA helps improve the surgical outcomes and GTR rates of ICDs at different sites.

## Introduction

Chondromas arise from dislocation of the embryonic tissue or transformation of the fibrous tissue. They can grow anywhere in the body where there is cartilage but most often occur in joint cartilage. Chondroma formation can be seen at the base of the skull due to intrachondral ossification at the embryonic stage, and residual chondrocytes can be present at the cartilaginous junction of the skull base sutures (1). Intracranial chondromas (ICDs), originally proposed and described by Hirschfield in 1851, are benign tumors composed of hyaline cartilage tissues that occur in the skull (2, 3). ICDs are mostly reported on a case-by-case basis, which makes correct diagnosis difficult and leads to a high rate of misdiagnosis (4, 5). To improve the diagnosis and treatment of ICDs, we reviewed a series of cases of admitted and surgically treated patients with ICDs and summarized their clinical manifestations, imaging features, surgical modalities, and pathological characteristics with the aim of improving the diagnosis and treatment of this type of tumor.

## Clinical Data and Methods

### Patient Data Collection

This study was approved by the Ethics Committee of the Tangdu Hospital of Air Force Medical University (AFMU; Xi’an, China) and the Mianyang Central Hospital (MCH; Mianyang, China). We reviewed 17 patients who underwent surgical treatment at the Tangdu Hospital of AFMU and at MCH between January 2010 and November 2021.

### Radiology Evaluation

Preoperative computerized tomography (CT), computerized-tomography angiography (CTA), and magnetic resonance imaging (MRI) were used to evaluate the sizes of ICDs and their relationships with surrounding nerves and blood vessels. Tumor volumes were calculated *via* MRI using the following formula:


$$\begin{array}{c} \text{Volume}=\text{maximum anteroposterior dimension (cm)}\times \text{maximum mediolateral}\\ \text{dimension(cm)}\times \text{maximum craniocaudal dimension (cm)/2} \end{array}$$

Imaging examination was reviewed by two experienced personnel, with a third person to evaluate if there was any disagreement between the two personnel.

### Surgical Approach and Intraoperative Auxiliary Equipment

All 17 patients had primary tumors and underwent one operation. Surgical approach was based on tumor location on MRI. The pterion approach was used to treat the lesions located in the sellar region and extending into the anterior and middle cranial fossa, while the orbitozygomatic approach was used to remove the tumors located in the parasellar region and extending into the middle and posterior cranial fossa and the upper slope. The subfrontal approach was mainly aimed at resecting anterior skull base tumors. The EETA was mainly applied for sellar tumors. For large tumors extending into suprasellar, parasellar and petroclival regions, the EEETA was required. In each patient undergoing EETA or EEETA, a nasal-septum mucosal flap was created to cover the dural defect. We used neuronavigation and Doppler to locate the internal carotid artery (ICA) and its branches during the operation. Occasionally, tumors were removed with the aid of a plasma knife.

### Assessment of the Extent of Resection

The extent of tumor resection was assessed on the basis of postoperative MRI and CT and was defined as follows: gross total resection (GTR), no tumor residue; subtotal resection (STR),>90% of tumor removed; and partial resection (PR), 50-90% of tumor removed.; and partial resection (PR), <90% of tumor removed.

### Systematic Literature Search

Our literature search was based on the Preferred Reporting Items for Systematic Reviews and Meta-Analyses(PRISMA) (6). We systematically searched reports in the English language from PubMed/MEDLINE and EMBASE databases since 1990 using the Boolean operators “OR” “AND” in combination OR with the following keywords: “chondromas,” “chondroma,” “intracranial,” “skull base,” “brain,” “sella,” and “sellar.” All identified articles were screened in two stages. Firstly, titles and abstracts were screened for relevant studies. Secondly, the full text was downloaded and assessed for eligibility. This process was carried out independently by three assessors. Any disagreements were resolved by consensus.

## Results

### Clinical Features

The clinical data of 17 patients for the ICDs are summarized in **Table 1**. Thirteen patients were female and four were male, ranging from 15 to 61 years of age (average, 33.7 years). The duration of clinical symptoms in patients ranged from 0.3 to 36 months (average, 8.6 months). Patients presented with varying clinical symptoms, including headache (5/17), dizziness (1/17), hypopsia (6/17), diplopia (3/17), facial numbness (2/17), ptosis (1/17), limb numbness (1/17), and sexual dysfunction (1/17; **Table 1**). Headache and hypopsia were the most common symptoms in the study. The possible cause of headache in five cases was elevated intracranial pressure (ICP) due to a large space-occupying lesion or the tumor irritating the dura mater. Hypopsia, diplopia, facial numbness and ptosis were caused by the tumor compressing the optic nerve, abducens nerve, trigeminal nerve and/or oculomotor nerve. Limb numbness was caused by the tumor compressing the medulla oblongata.

**Table 1:** Seventeen patients treated for intracranial chondroma between January 2010 and November 2021.

Case #	Sex	Age (y)	Symptoms	Symptom duration (m)	Tumor site	Tumor volume (cm^3^)	KPS (Pr)	Preoperative diagnosis	Surgical procedure	Tumor texture	Postoperative symptoms/relief	KPS (departing hospital)	Follow-up (m)
1	F	50	Face and limb numbness	36	Medulla	8.93	80	Chordoma	Posterior midline	Hardness	Symptoms disappeared	80	60
2	F	49	Headache; hypopsia	1	Middle cranial fossa, parapharyngeal space, jugular foramen, pons	216.55	60	Cavernous hemangioma	Orbitozygomatic	Hardness	Headaches disappeared; vision improved; subcutaneous hydrops; ICI	70	24
3	F	23	Hypopsia	0.3	Sella	9.98	90	Pituitary adenoma	EETA/EEETA	Toughness	Vision improved	90	60
4	F	45	Headache	2	Suprasellar cistern, prepontine cistern, cisterna cruralis	30.97	80	Chondroma	EETA/EEETA	Hardness	Headache relief; CSFL; ICI	80	60
5	F	36	Facial numbness; hypopsia	36	Cavernous sinus, slope, sella, pharyngeal recess	192.86	70	Cavernous hemangioma	Orbitozygomatic	Hardness	Facial numbness improved; no change in vision	80	36
6	F	29	Headache	2	Sella, parasellar, slope, middle cranial fossa, hippocampus	229.15	60	Chondroma	Orbitozygomatic	Hardness	Diplopia; left-limb hemiplegia; pulmonary infection; ICI	40	60
7	F	15	Diplopia	6	Parasellar, petroclival regions	36.26	80	Chondroma	EETA/EEETA	Hardness	Diplopia	80	48
8	F	21	Diplopia	3	Cavernous sinus	11.50	90	Chondroma	EETA/EEETA	Hardness	Blepharoptosis; diplopia	80	48
9	F	61	Dizziness	1	Anterior skull base	2.22	90	Chondroma	Subfrontal	Hardness	—	90	48
10	M	25	Hypopsia	2	Sella	40.96	80	Pituitary adenoma	EETA/EEETA	Hardness	Vision improved	90	48
11	F	23	Blepharoptosis	1	Sella	11.02	90	Meningioma	EETA/EEETA	Hardness	Blepharoptosis	90	36
12	M	21	Diplopia	2	Sella	6.91	90	Pituitary adenoma	EETA/EEETA	Toughness	Diplopia	90	24
13	F	24	Hypopsia	2	Middle cranial fossa	18.4	90	Schwannoma	EETA/EEETA	Toughness	Diplopia; no change in vision	80	24
14	M	30	Sexual dysfunction	24	Sella	12.83	90	Craniopharyngioma	EETA/EEETA	Hardness	Sexual dysfunction	90	12
15	F	36	Headache	1	Parasellar	11.66	90	Meningioma	Pterion	Hardness	Headache relief	80	60
16	F	44	Headache	3	Anterior skull base	8.69	90	Meningioma	Subfrontal	Hardness	Headache relief	90	3
17	M	42	Hypopsia	24	Cavernous sinus, middle cranial fossa	27.55	80	Meningioma	Pterion	Hardness	Vision improved	80	36

y, year; m, month; Pr, preoperative; CSFL, cerebrospinal-fluid leakage; ICI, intracranial infection; EETA, endoscopic endonasal transsphenoidal approach; EEETA, extended endoscopic endonasal transsphenoidal approach.

### Preoperative Imaging and Diagnosis

Preoperative imaging indicated that all lesions were located in the skull base. Details of tumor invasion sites are shown in **Table 1**. The range of tumor invasion included the anterior skull base (2/17), sella (9/17), parasellar region (7/17), middle cranial fossa (4/17), medulla oblongata (1/17), and slope (3/17). The average tumor volume was 51.55 cm^3^ (range, 2.13–229.15 cm^3^). Cranial CT scans of the 13 patients showed clear boundaries between lesions and brain tissue, and calcification foci were observed in all lesions. We found patchy calcification in 10 patients, marginal calcification in 3, bone destruction in 2, and hypodensity shadow in 1. Additional CTA or MRA was performed in five patients to determine the relationships between tumors and surrounding blood vessels. On MRI, lesions were clearly demarcated from the surrounding brain tissue, and there were mixed signals with isometric or long T1 and long T2 signals, but the calcified part showed long T1 and short T2 signals. MRI enhancement showed uneven enhancement. According to preoperative-imaging findings, one case was diagnosed with craniopharyngioma, one with chordoma, one with schwannoma, two with cavernous hemangioma, three with pituitary tumor, four with meningioma, and five with chondroma (**Table 1**).

### Surgical Outcomes

We selected surgical approaches based on tumor size and location with minimal damage to brain tissue (**Table 2**). Tumors were removed by EETA/EEETA in nine cases and craniotomy in eight. The surgical approaches for craniotomy mainly involved the posterior midline approach (1/17), orbitozygomatic approach (3/17), pterion approach (2/17), or subfrontal approach (2/17). Intraoperatively, tumors were found to be tightly adherent to the ICA (11/17) and to cranial nerves (CNs) II (6/17), III (6/17), V (4/17), and VI (4/17). Most tumors were located in the epidural space, but some had eroded into the subdural space. All lesions were closely associated with the skull and were milky white in color, and most clearly contained cartilage-like debris.

**Table 2:** Extent of tumor resection.

	GTR	STR	PR	Total
EETA/EEETA	8	1	0	9
Posterior midline	0	1	0	1
Orbital zygomatic	0	2	1	3
Pterion	0	1	1	2
Subfrontal	2	0	0	2
Total	10	5	2	17

GTR, gross total resection; STR, subtotal resection; PR, partial resection; EETA, endoscopic endonasal transsphenoidal approach; EEETA, extended endoscopic endonasal transsphenoidal approach.

In this group, 10 cases were completely resected, 5 were subtotally resected, and 2 were partially resected (**Table 2**). During assessment 1 week after surgery, facial and limb numbness on the right had disappeared in one case, facial numbness had improved in one case, but the patient had no improvement in visual acuity. Dizziness was relieved in one case, visual acuity had improved in three cases, headache was relieved in four cases, sexual function had no improvement in one case, and diplopia was unimproved in three cases.

### Postoperative Complications

Postoperative complications mainly included diplopia combined with right-limb hemiparesis (1/17), diplopia (2/17), eyelid ptosis (1/17), pulmonary infection (1/17), subcutaneous hydrops (1/17), cerebrospinal fluid leakage (CSFL; 1/17), and intracranial infection (ICI; 3/17; **Tables 1**, **3**). ICI was cured by continuous lumbar drainage and antibacterial treatment, and CSFL was cured by bedrest and continuous lumbar-cistern drainage. Subcutaneous fluid was cured by puncture and pressure bandaging. The case of pulmonary infection was cured by physical intervention and antibiotic treatment.

**Table 3:** Surgical morbidity.

Postoperative morbidity	Surgical morbidity at 1 week	Permanent surgical morbidity
CN III	1	1
CN VI	2	2
Sexual dysfunction	1	0
Pulmonary infection	1	0
Subcutaneous hydrops	1	0
CSFL	1	0
ICI	3	0
Hemiplegia	1	0
Tumor recurrence and death	0	2

CN, cranial nerve; CSFL, cerebrospinal-fluid leakage; ICI, intracranial infection.

### Histopathology and Immunohistochemistry

Pathological examination showed that the tumors consisted of differentiated cartilages, with or without varying amounts of ossification, calcification, fibrogenesis, or mucus formation. Pathology showed a trend of chondrosarcoma transformation in three cases. In order to distinguish chondroma from other tumors, immunohistochemistry (IHC) was performed in 10 cases and showed positive for S100 and Vimentin in 10 cases, Ki-67 ≤ 3% in 3 and Ki-67 >3% in 2, positive for SOX-9 in 1, and negative for epithelial-membrane antigen (EMA), cytokeratin (CK), CK18, CD34 and glial fibrillary acidic protein (GFAP) in 10 cases.

### Follow-Up

In this study, we evaluated clinical symptoms and imaging during outpatient visits or over the phone at 3 months, 6 months, and every year for 5 years after 6 months. Follow-up duration range was 3–60 months (average, 40.41 months). One patient received gamma knife treatment 3 months after surgery, while the remaining patients received no adjuvant therapy. Two patients died due to progression of tumor recurrence (including the patient who received postoperative radiotherapy). None of the remaining patients had tumor recurrence.

### Systematic Review

**Figure 1:**
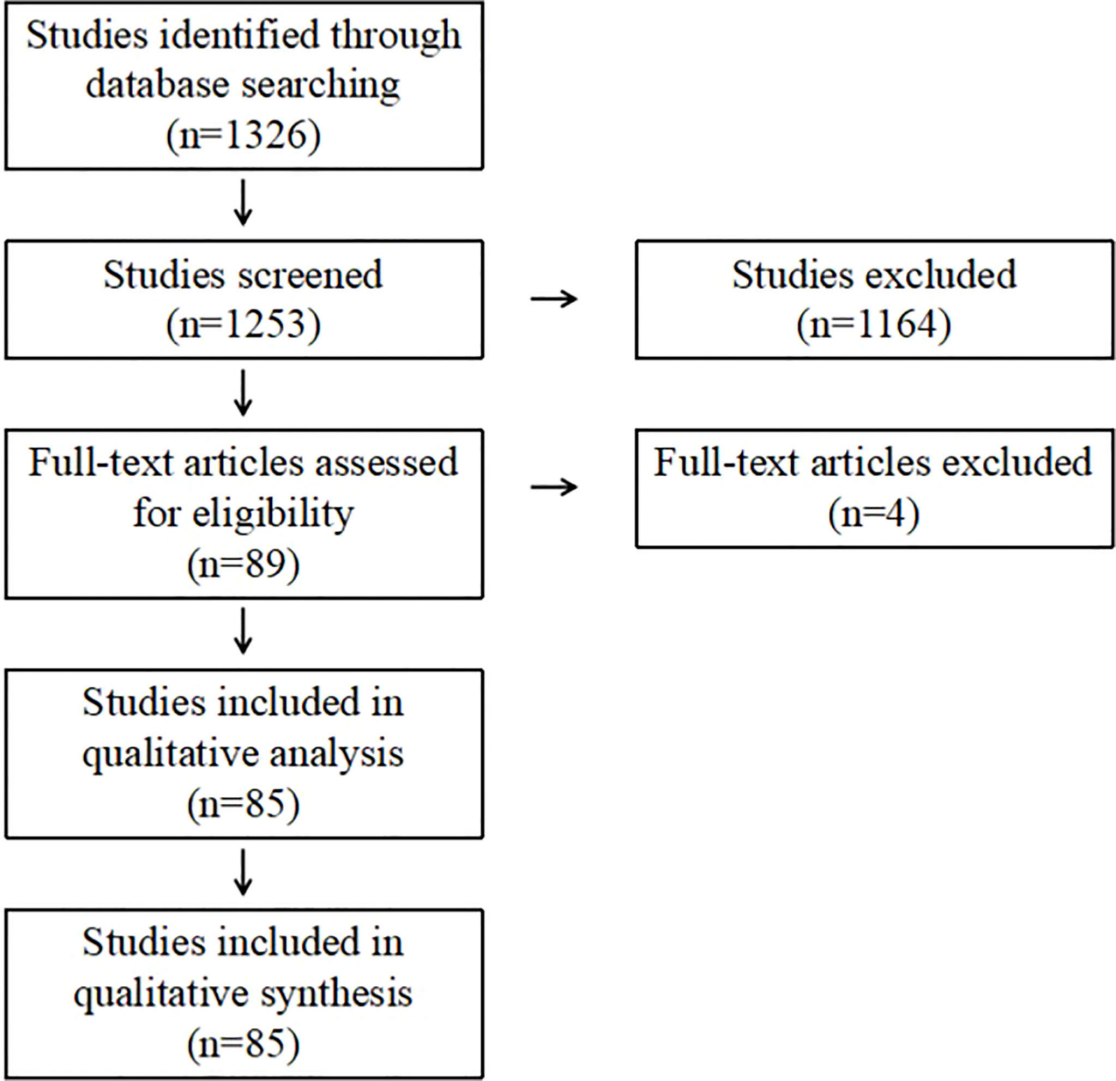
Flow chart outlining literature search using PRISMA.

Seventy-three duplicate records were removed through database search, leaving a total of 1253 records available for title and abstract checking. Eighty-nine full-text articles were evaluated. Finally, 85 articles were included for analysis (**Figure 1**).

All articles were predominantly case reports and included 164 cases. The proportion of ICDs was found to be approximately equal in men (n=79) and women (n=85), with a minimum age of 8 years and a maximum age of 73 years (mean 32.6 years). The clinical manifestations of ICDs mainly included headache (n=62), oculomotor paralysis (n=24), abducent paralysis (n=18), and epilepsy (n=18). ICDs primarily appeared on MRIs as irregular long T2 signal with punctate low T2 signal and a few of them containing long T1 and long T2 signals (7, 8). CT scans showed calcification in almost all tumors. ICDs were mainly distributed in skull base (n=87), convexity (n=30), intraparenchymal (n=25), falx cerebri (n=14), and others (n=8). The majority of cases were misdiagnosed as meningioma or low-grade glioma before surgery. All patients underwent surgical treatment, and almost all ICDs in convexity, intraparenchymal, and falx cerebri were gross-totally removed.

All chondromas in skull base were removed by craniotomy except for seven cases in which chondromas were removed by EETA, including GTR (n=18), STR (n=34), and PR (n=31). Extent of tumor resection was not recorded in four cases of chondromas in skull base, and one case died during hospitalization after craniotomy. Nine of all postoperative patients received radiotherapy.

On histopathology, ICDs usually presented as irregularly sized mature chondrocytes with calcification and fibrous components. Based on IHC, S-100 and vimentin were positive, and cytokeratin, epithelial membrane antigen, and Ki67 were negative.

## Case illustration

### Case 1

**Figure 2:**
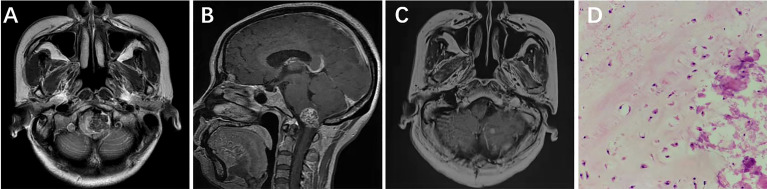
Left side of the medulla oblongata showed a round mixed short T2 signal shadow within which speckled long T2 signal was seen **(A)**. Sagittal contrast-enhanced MRI showed that medulla oblongata was compressed by confounding signal mass located in foramen magnum **(B)**. Six months after surgery, enhanced MRI showed that the tumor had not recurred **(C)**. Pathological results at 200× magnification showed mature chondrocytes of different sizes and vacuolar formation in some cells **(D)**.

A 50-year-old female presented with left-facial and right–lower-extremity numbness combined with choking and difficulty swallowing that had lasted 3 years. Physical examination showed hypoesthesia in the right-lower extremity; the rest of the neurological examination was negative. Head CT indicated a high-density shadow (about 2.5 cm × 1.7 cm) on the left side of the medulla oblongata, reaching up to the bottom of the fourth ventricle, protruding down into the foramina magnum, and displacing the medulla oblongata to the right due to compression. Cranial MRI scan showed isotonic T1 signal with mixed long T2 signal and speckled short T2 signal changes within the tumor (**Figure 2A**); the enhanced scan showed inhomogeneous enhancement (**Figure 2B**). Preoperative diagnosis was chordoma. A posterior midline approach was performed intraoperatively. The lesion was white, hard as bone and mostly located in the epidural space, with some erosions protruding into the dura and closely adhering to the medulla oblongata. We carefully separated the tumor along the medulla oblongata with a stripper, and total tumor resection was achieved under microscopic guidance. Postoperative MRI suggested there was no residual tumor in the surgical area (**Figure 2C**). The pathological diagnosis was chondroma (**Figure 2D**); ICH showed Vimentin and S-100 stain was positive, but Ki-67, CK, CK18, EMA, CD34 and GFAP stain was negative. After surgery, the numbness on the left side of the patient’s face and in her right-lower extremity disappeared, as did her coughing on swallowing. There was no tumor recurrence during 60 months’ follow-ups.

### Case 2

**Figure 3:**
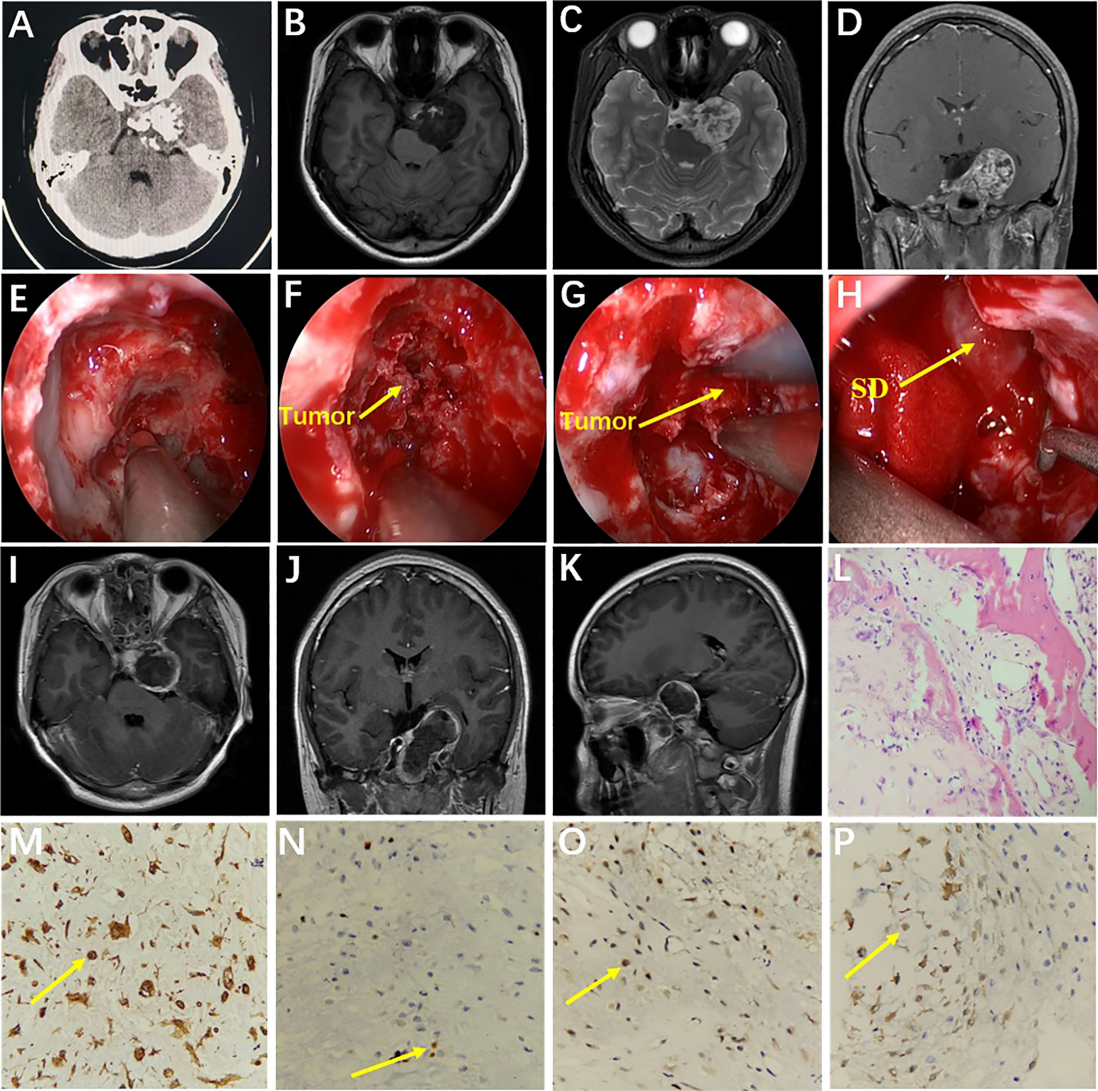
Preoperative CT showed medium-high mixed density in the sella **(A)**. Preoperative MRI showed long T1 **(B)** and long T2 **(C)** signals in the left parasella. Multiple patchy short T2 signals were seen in the lesion **(C)**. After enhancement, the lesion showed heterogeneous enhancement **(D)**. Intraoperative drilling of anterior wall of sphenoid sinus and sella floor by EEETA **(E)**. Resection of tumor located in sella turcica **(F)**. Resection of tumor in the left parasella **(G)**. Descent of sellar diaphragm after removal of tumor **(H)**. Postoperative axial, coronal, and sagittal MRI enhancement showed that the tumor was completely removed and there was no residual tumor in surgical area **(I–K)**. Histopathological examination (hematoxylin and eosin [H&E] staining) at 200× magnification showed tumors in the myxoid matrix background consisting of hyaline chondrocytes with irregular lobules, spindle cells, and chondrocytes with homogeneous micronuclei **(L)**. Immunohistochemical staining at 400× magnification showed vimentin (+, **M**), Ki-67 (6%, **N**), SOX-9 (+, **O**), and S-100 (+, **P**). EEETA, extended endoscopic endonasal transsphenoidal approach; SD, sellar diaphragm.

A 15-year-old female was admitted with diplopia that had lasted 6 months. She had a history of left–upper-extremity and right–lower-extremity fractures. On physical examination, the left eye was limited in abduction; the remaining neurological examination was negative. Cranial CT scan showed abnormal mixed density in the sellar and parasellar regions (**Figure 3A**). Cranial MRI scan suggested a long T1 signal and a long T2 signal in the parasellar region and the anterior and lateral pons, and multiple patches of short T1 and short T2 signal were found in the lesion (**Figures 3B, C**). After enhancement, the lesion showed heterogeneous enhancement (**Figure 3D**). The preoperative diagnosis was chondroma. Surgery was performed *via* the EEETA, creating the nasal septal mucoperiosteal flap first. Then, a grinding drill was used to remove the bone, including the anterior wall of sphenoid sinus, sellar floor, and part of slope and the anterior clinoid process, in order to expose the left cavernous sinus. Neuronavigation and Doppler helped clarify the tumor boundary, the location of the ICA and the extent of bone abrasion. We resected a portion of the tumor using a 0° scope and then probed toward the left petrous apex to resect the remaining tumor using a 30° scope. Neuronavigation aided in removing tumors adjacent to the anterior and lateral brainstem. Intraoperatively, the lesion was found to be grayish red in color, hard in texture, located in the epidural and partially closely adherent to the dura mater (**Figures 3E–H**). Postoperative MRI suggested GTR of the tumor (**Figures 3I–K**). On histopathological examination, the tumor was diagnosed as chondroma (**Figure 3L**); Vimentin (**Figure 3M**), Ki-67 (6%) (**Figure 3N**), SOX-9 (**Figure 3O**), and S-100 (**Figure 3P**) stained positive, but CK, CK18, EMA, CD34 and GFAP stained negative. The patient had no immediate improvement in diplopia or left-eye abduction restriction after surgery. The diplopia improved 3 months after surgery. No recurrence was observed during 60 months’ follow-ups.

### Case 3

**Figure 4:**
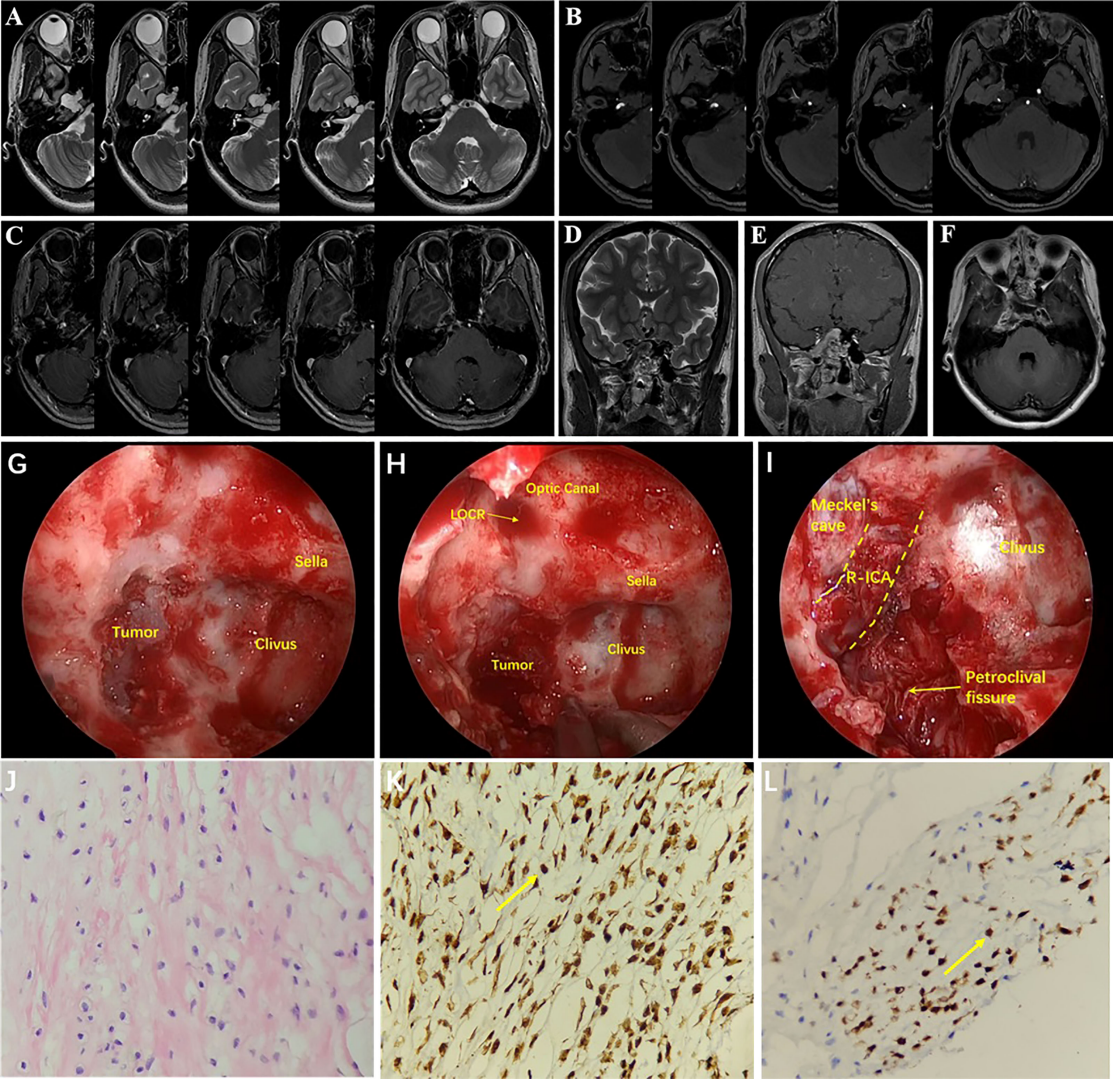
MRI showed irregular long T2 signal and punctate low T2 signal at the base of the right-middle cranial fossa and posterior cranial fossa **(A)**. The tumor communicated intracranially and extracranially. MRA showed compression and displacement of the right ICA by the tumor **(B)**. The tumor showed uneven enhancement **(C)**. Coronal T2 image **(D)** and coronal T1 **(E)** and axial T1 **(F)** contrast-enhanced images on postoperative MRI showed that the tumor was resected completely. Drilling the petroclival bone and exposing the tumor **(G)**. Resection of petroclival tumor **(H)**. After resection of tumor, right internal carotid artery and petroclival fissure were exposed **(I)**. Histopathology results at 200× magnification revealed chondrocytes of varying sizes and cytoplasmic intermediate vacuole formation **(J)**. Immunohistochemical staining at 400× magnification showed vimentin (+, **K**) and S-100 (+, **L**). LOCR, lateral opticocarotid recess; R-ICA, right internal carotid artery.

A 24-year-old female had hypopsia in her left eye for 2 months. On physical examination, visual acuity of the left eye decreased; the remaining neurological examination was negative. Cranial MRI showed the tumor with irregular long T2 signal and dotted low T2 signal (**Figure 4A**), and the enhanced scan showed inhomogeneous enhancement (**Figure 4C**). The tumor communicated intracranially and extracranially. Magnetic resonance angiography (MRA) showed compression and displacement of the right ICA of the petrous and the cavernous segments (**Figure 4B**). Preoperative diagnosis was schwannoma. Surgery was performed by the EEETA, creating the septal mucoperiosteal flap first. After removing the bone of the anterior wall of sphenoid sinus and the posterior part of nasal septum, optic nerve, and ICA prominence, clival recess was exposed. Then, upon continuously grinding the petrous bone, the dura remained intact and then was incised by a hook knife. Hard white tumor tissue, partially protruding into the subdural space, was visible and was resected piecemeal (**Figures 4G–I**). Postoperatively, the patient had complications of limited abduction of the right eye and diplopia, and no improvement in left-eye visual acuity. Postoperative MRI suggested GTR of the tumor (**Figures 4D–F**). On histopathological examination, the tumor was diagnosed as chondroma (**Figure 4J**); Vimentin (**Figure 4K**), S-100 (**Figure 4L**) and Ki-67(2%) stained positive, but EMA, CK, CK18, CD34, SOX-9 and GFAP stained negative by ICH. Diplopia, right-eye abduction restriction and visual acuity improved 3 months after surgery. Six months after surgery, diplopia and right-eye abduction restriction had returned to normal. There was no recurrence during 24 months’ follow-ups.

## Discussion

ICDs are very rare benign tumors of the central nervous system that can occur alone or as part of Ollier disease or Maffucci syndrome (9). In our study, no case had Ollier disease or Maffucci syndrome. Isolated chondromas usually occur at the cartilaginous junction at the base of the skull, most commonly in the sellar and parasellar regions, and some invade the middle cranial fossa or brainstem (10). In addition, some chondromas have been reported in other areas such as the endocranium and cerebral parenchyma (11–13). Our study reported chondromas involving the sella, parasellar region, and middle cranial fossa, as well as a rare case of chondroma located adjacent to the medulla oblongata and with intracranial and extracranial communication. Based on the findings of this study and previous ones, the onset age of chondroma ranges from 15 months to 60 years (14). Due to the tumor’s slow growth, it is often not detected until it is large and causes symptom onset. Common symptoms are headache and neurological deficits (15). In this study, we reported patients aged 15–60 years with an average age of 33.7 years, whose main clinical manifestations were headache and focal neurological deficits, including the palsy of the optic, abducens, trigeminal, and oculomotor nerves.

The incidence of chondroma is low, and its clinical manifestations and imaging features are similar to those of pituitary adenomas, craniopharyngioma, meningioma, cavernous hemangioma, chondrosarcomas, and chordoma. Thus, distinguishing chondroma from other space-occupying lesions is difficult, and preoperative diagnosis for chondroma is important for selecting surgical methods. CT imaging of chondromas usually shows lobulated high-density shadow with patchy calcification inside the tumor. It also shows calcification at the edge of the tumor with or without erosion of the surrounding bone (16, 17). Some tumors show low density due to necrosis or cystic changes (16, 17). On T2-weighted MRI, chondromas exhibit mixed hypersignal, while calcification on T2 sequence shows low signal (18). MRI shows inhomogeneous enhancement after intravenous injection of gadolinium. Due to the slow growth of chondromas, no peritumoral edema appears on MRI. It has also been reported that the cartilage matrix in chondroma can lead to its low signal on diffusion-weighted imaging (DWI), and the arrangement of cells in the lesion shows the apparent diffusion coefficient (19, 20). The imaging characteristics, including the “snowman” shape of pituitary tumor, “dural tail sign” of meningioma, vascular lumen and thrombus of cavernous hemangioma, and eggshell calcification of craniopharyngioma, could distinguish ICDs from other tumors (21–23). As a type of chordomas, chondroid chordomas grow slowly but have a higher incidence of bone destruction than chondromas due to aggressive behavior toward the axial skeleton (24). Chondrosarcomas present as osteolytic destruction on CT. MRI showed that chondrosarcomas are composed of lobules that show high signal on T2. After MRI enhancement, the periphery of most lesions is significantly enhanced, while the lobules themselves are generally not enhanced, and heterogeneous ring, bow, or septal enhancement is seen within the lesions. The distribution, length, diameter, and thickness of the annular, arcuate, or septal enhancement are correlated with the malignancy of chondrosarcoma (25). Of the 17 cases in our study, 12 were preoperatively misdiagnosed as craniopharyngioma, chordoma, cavernous hemangioma, pituitary adenomas, and meningioma, and 5 were preoperatively diagnosed as chondroma. MRI combined with CT examinations were performed in all five cases diagnosed with chondromas.

Chondromas tend to be irregular white masses with a bone-like texture. Cartilage ossification occurs around chondromas and is often accompanied by calcification. Microscopically, tissues are calcified with disorganized structures and varying cell sizes; the cells are similar to normal cells with hyaline chondrocytes (26). Uncalcified tissues are mostly composed of mesochondrium and cartilaginous stroma containing thin-walled blood vessels. Microscopic chondroma usually do not have cellular atypia or mitosis, whereas increased cellularity, infiltrative growth, cellular atypia, >20% myxoid changes, mitosis, and pleomorphism suggest chondrosarcoma (15, 27, 28). Chondromas are reported to be positive for vimentin and S-100 protein and chordomas for CK, EMA, S-100 protein, vimentin and brachyury; these are pathological discrimination indexes between these two types of tumors (4, 15). Brachyury that plays a key role in the notochord formation is expressed in chordomas, but it cannot be used as a sensitive indicator of prognosis (29).

For patients with ICDs, surgical treatment is the most common and effective modality after onset of clinical symptoms. However, due to the growth characteristics of chondromas, they are usually found when growing to a large size and causing clinical symptoms. The tumor compresses or encompasses the blood vessels and nerves at the base of the skull, so the difficulty and risk of total tumor resection have increased remarkably. To avoid serious postoperative complications, the tumor can be subtotally or partially removed to alleviate clinical symptoms (30).

With the development of endoscopic equipment and various extended endoscopic approaches, as well as the accumulation of experience for neurosurgeons, endoscopic resection of sellar and parasellar tumors has made great progress. When different craniotomy approaches were used for chondromas excision in the anterior, middle, and posterior cranial fossa (12, 31), there were also a few retrospective reports about endoscopic tumor resection for chondromas in the sellar or parasellar region (32). When the tumor is located in convexity or intraparenchymal, craniotomy is implemented under the microscope. When the tumor is located in the midline or paramidline region of the skull base, operation is generally performed *via* EETA/EEETA. When the tumor grows from the midline or paramidline region to anterior, middle or posterior cranial fossa beyond the endoscopic field of view, endoscopic transnasal sphenoidal surgery combined craniotomy may need to be performed. Because chondromas mostly originate from cartilage remnants at the base of skull, they are usually located in the epidural space (11). Thus, for chondromas in the sellar and parasellar areas, especially for those with intracranial and extracranial communication, ICDs can be resected by the EETA/EEETA, while neuronavigation and Doppler can help avoid damage to neurovascular. Previous studies reported that the major surgical procedure for ICDs was craniotomy, and the GTR rate was about 22.7–36.7% (12, 15). In our study, the GTR rate was 58.8%, significantly higher than 36.7%. Furthermore, a satisfactory outcome was obtained by the EETA/EEETA, and 9 of 17 cases were resected in this way, one with STR and the remaining eight with GTR.

The author summarize the advantages of the EETA/EEETA for chondroma resection as follows: 1) ICDs are mostly located in the midline or paramidline regions of the skull base, which can be resected with broad surgical field by endoscope; 2) ICDs are usually located in the epidural space, some of which invade into the subdural space. By grinding the bone of the anterior skull base, sella, petroclival region or foramen magnum, the tumor is easily exposed and removed; 3) Surgery is performed in the epidural area to help protect cranial nerves and the brain tissue from damage; 4) Transnasal endoscopic surgery has less trauma and quick recovery compared to craniotomy. However, for chondromas that invade the subdural space and compress and encompass the ICA and CN, it is still difficult to remove the tumor completely. The neurosurgeons need to carefully identify the ICA and its branches and intracavernous CN to avoid injury to these structures. One complication of transnasal endoscopic surgery is CSFL. For patients with CSFL caused by intraoperative dural rupture, a close repair is required after tumor resection, which is performed by covering the dural defects using fascia lata and fat, nasal septal mucoperiosteal flap, and biological glue. In our patients, there were no injury of ICA and no occurrence of postoperative CSFL.

Whether patients need to receive radiotherapy after surgery was still controversial, and it has been shown that radiation can increase the risk of recurrence (17, 33). Furthermore, partial resection followed by radiotherapy has been reported to result in malignant transformation of the tumor into chondrosarcoma (17, 33). Thus, radiotherapy is not recommended for patients with remnant chondromas. In our study, two cases recurred postoperatively, which might have been related to mitotic activity, radiotherapy, and/or degree of tumor resection.

### Limitations of This Study

This study was designed retrospectively and could not avoid the common biases that could have been avoided in controlled studies. In our study, the average time of follow-up was 40.41 months, which might require a longer duration to observe whether there was a recurrence of tumor after surgery. Additionally, due to the extremely low incidence of ICDs, further statistical evaluation was not done owing to a small number of cases.

## Conclusions

ICDs lack typical imaging features and are often misdiagnosed as other tumors. Simultaneous CT and MRI can improve the diagnostic rate of chondromas. The final diagnosis must be confirmed by histopathology. The EETA or EEETA helps improve surgical outcomes and increase the GTR rate of chondromas in the sella, parasellar region, and of those even extending into the middle cranial fossa and petroclival regions. The use of intraoperative neuronavigation and Doppler can help distinguish surrounding blood vessels and nerves. 

## Data Availability Statement

The original contributions presented in the study are included in the article/supplementary material. Further inquiries can be directed to the corresponding authors.

## Author Contributions

Investigation: HL,YX and YZ Methodology: HL and QC. Project administration:TZ and YW Resources: HL and YW Supervision: TZ and YW Histopathological examination and ICH: JL and ZL Original draft: HL and YW Review & editing: HL and YW. All authors contributed to the article and approved the submitted version.

## Funding

This work was supported by from the National Natural Science Foundation of China (No. 81971153) and the Foundation Program of Sichuan Provincial Health Commission (No. 21PJ181).

## Conflict of Interest

The authors declare that the research was conducted in the absence of any commercial or financial relationships that could be construed as a potential conflict of interest.

## Publisher’s Note

All claims expressed in this article are solely those of the authors and do not necessarily represent those of their affiliated organizations, or those of the publisher, the editors and the reviewers. Any product that may be evaluated in this article, or claim that may be made by its manufacturer, is not guaranteed or endorsed by the publisher.

## Abbreviations

GTR, gross total resection; STR, subtotal resection; PR, partial resection; EETA, endoscopic endonasal transsphenoidal approach; EEETA, extended endoscopic endonasal transsphenoidal approach; CN, cranial nerve; CSFL, cerebrospinal fluid leakage; ICI, intracranial infection; ICA, internal carotid artery; IHC, immunohistochemistry.
